# Melanoma of Unknown Primary Presenting as a Renal Mass: A Case Report

**DOI:** 10.7759/cureus.105128

**Published:** 2026-03-12

**Authors:** Shelby Baxter, Zachary Aguilar, Gabrielle Yankelevich, Laura Spruill, Matvey Tsivian

**Affiliations:** 1 Medical School, Kansas City University of Medicine and Biosciences, Joplin, USA; 2 Department of Urology, Medical University of South Carolina, Charleston, USA; 3 Pathology, Medical University of South Carolina, Charleston, USA

**Keywords:** melanoma metastatic, melanoma of unknown primary, melanoma treatment, robotic partial nephrectomy, small renal mass, surgical case reports

## Abstract

Melanoma of unknown primary (MUP) is a rare presentation of melanoma most commonly found in the lymph nodes, subcutaneous tissue, and visceral organs. Renal involvement has rarely been demonstrated in the literature. We reported a 66-year-old male with an incidental finding of a small (1.7cm) right renal mass who underwent partial nephrectomy with pathology consistent with metastatic melanoma. After thorough evaluation, no primary melanotic lesion was identified, therefore characterizing his disease as MUP. The patient subsequently was treated with ipilimumab and nivolumab and is continuing on maintenance therapy with nivolumab. This case is one of three known cases of MUP presenting as a renal mass according to current literature. Though rare, this highlights the importance of including MUP in the differential diagnosis of renal masses.

## Introduction

A majority of melanoma cases are diagnosed with a known primary site, most commonly the skin [1]. Melanoma is predominantly diagnosed as an integumentary lesion; however, when there is no primary lesion identified, it is referred to as melanoma of unknown primary (MUP). 

MUP is rare and accounts for approximately 3.2% of melanoma diagnoses [1]. Most common sites of diagnosis are lymph nodes (LNs), followed by subcutaneous sites and lastly visceral organs [1]. Rarely has MUP been discovered as a renal mass, with very few cases reported. Renal metastasis is a common site for metastatic melanoma with a known primary lesion, along with a multitude of solitary masses often found at autopsy of metastatic melanoma patients [2]. We report a case of MUP in the kidney, which was treated with right partial nephrectomy and adjuvant systemic therapy consisting of ipilimumab and nivolumab. Per literature review, this is the third case of melanoma presenting as a renal mass without a known prior history of melanoma reported in the literature [2,3].

## Case presentation

**Figure 1:**
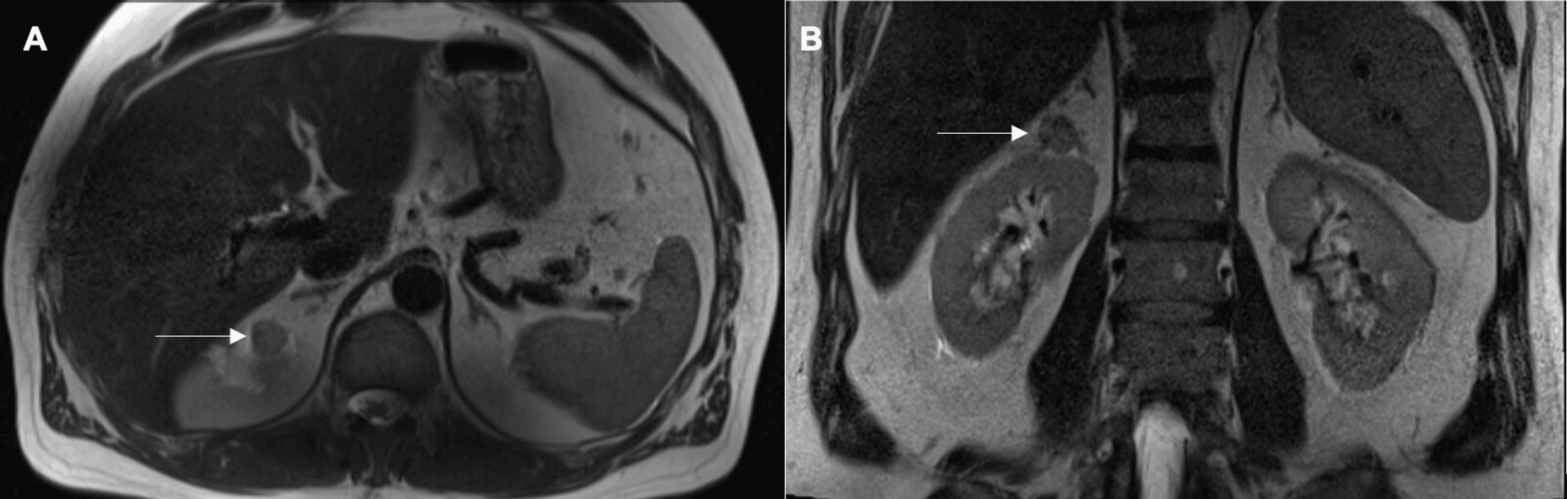
MRI of right renal mass. (A) Axial MRI demonstrating a 1.7cm exophytic lesion arising from the right superior renal pole (white arrow). (B) Coronal MRI demonstrating the same lesion.

A 66-year-old man with a history of basal cell carcinoma and a remote pulmonary embolism on Xarelto underwent CT imaging of the abdomen and pelvis for evaluation of weight loss and gastrointestinal symptoms, which incidentally revealed an 8-mm indeterminate lesion in the superior pole of the right kidney. Three-month interval MRI demonstrated a 1.7 cm exophytic right superior pole renal lesion on cross-sectional imaging (Figure 1).

**Figure 2:**
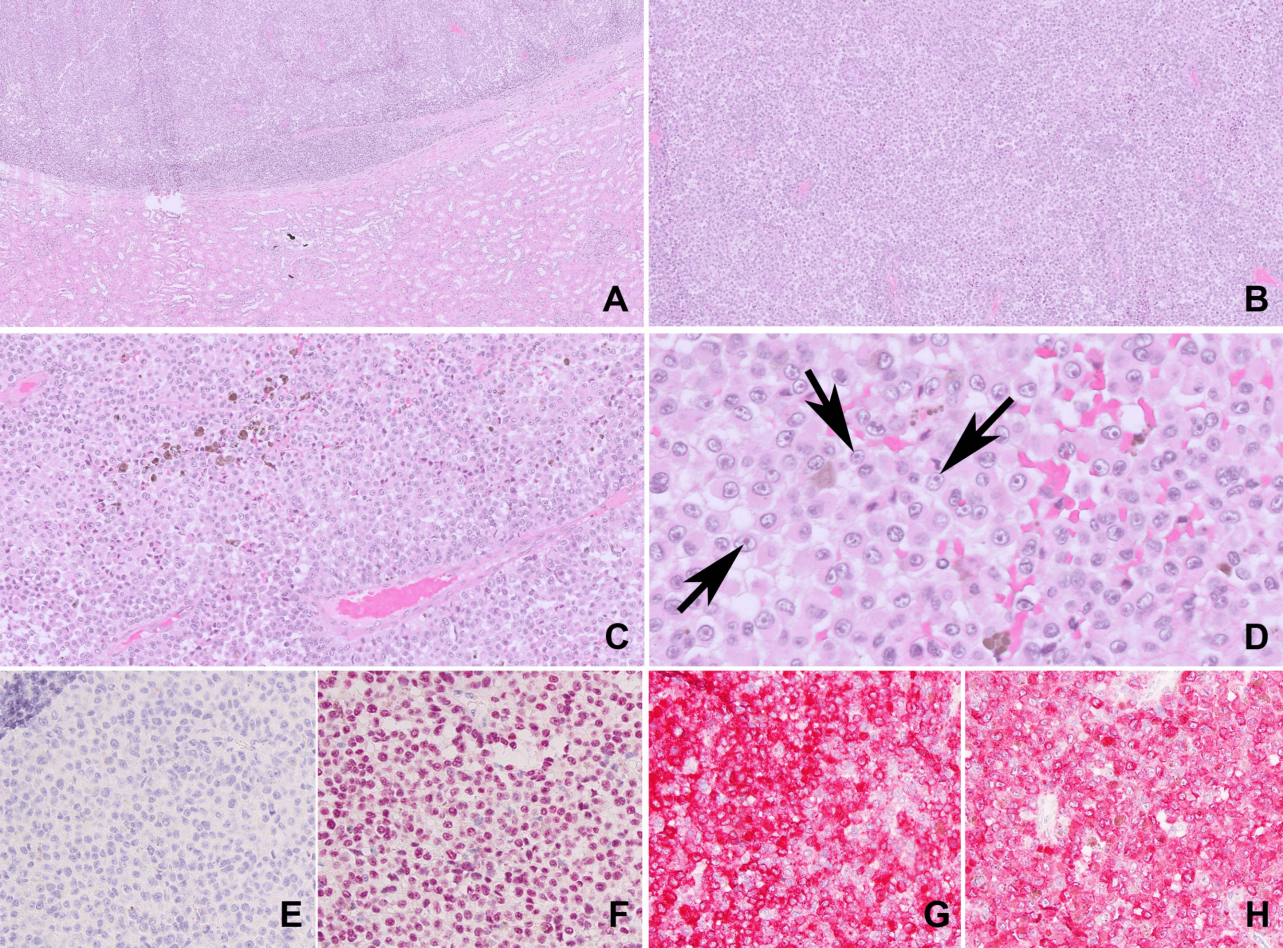
Histopathologic features of renal melanoma of unknown primary. Hematoxylin and Eosin Slides. (A) 5x magnification demonstrating malignant melanoma abutting but not invading kidney parenchyma. (B) 10x magnification demonstrating sheets of cells with relatively uniform nuclei, prominent nucleoli with scattered single cell necrosis and apoptotic bodies. (C) 20x magnification demonstrating melanin pigment scattered throughout the tumor. (D) 60x magnification demonstrating the characteristic “cherry red” nucleoli characteristic of melanoma (marked by arrows). (E) 20x magnification of negative pan-keratin immunohistochemical stain. (F) 20x magnification of positive Sox-10 immunohistochemical stain. (G) 20x magnification of Mart-1 Immunohistochemical stain. (H) 20x magnification of HMB45 immunohistochemical stain. The combination of the histology, negative keratin staining and positive melanoma markers (Sox-10, Mart-1 and HMB45) support the diagnosis of malignant melanoma.

He had no complaints of hematuria or flank pain. His physical exam was non-contributory, and laboratory results were within normal limits. He underwent an uncomplicated robotic-assisted right partial nephrectomy with pathology consistent with metastatic melanoma. The patient received a second opinion at another cancer center for personal reasons, where they concurred with the diagnosis and provided guidance with treatment. There, his histopathology slides were reviewed and confirmed metastatic melanoma (Figure 2). He underwent an extensive workup to identify a primary lesion, including evaluations with dermatology, ophthalmology, and urology, along with random bladder and prostatic urethral biopsies, which were all negative. Therefore, the patient was diagnosed with MUP. He has completed four cycles of ipilimumab and nivolumab and is continuing on maintenance therapy with nivolumab with plans for one year of therapy. A recent PET scan demonstrates no evidence of disease. 

## Discussion

The definitive cause of MUP remains unknown since it was described in 1963 by Das Gupta, who was the first to classify MUP [4]. Suggested by previous cases of MUP, a thorough skin evaluation followed by subungual otorhinolaryngologic and ophthalmologic examination is recommended for workup. After biopsy, the specimen requires histological confirmation by immunohistochemical stains with S100, vimentin, and Human Melanoma Black 45 (HMB-45). A computed tomography (CT) scan of the thorax, abdomen, and pelvis, along with magnetic resonance imaging (MRI) of the brain, is also required to determine if additional lesions are present [2]. In terms of tumor staging, MUP that has been identified on visceral organs is diagnosed as stage IV disease, per the eighth edition of the American Joint Committee on Cancer (AJCC) staging manual [5].

To our knowledge, only two other cases of MUP presenting as a renal mass have been reported (Table 1). In one case, a 67-year-old male presented with right flank pain with concomitant microscopic hematuria. The skin and ophthalmologic examinations were negative for an occult primary tumor, whereas the ultrasonographic examination revealed a hypoechoic mass in the lower pole of the right kidney. After surgical resection, the tumor cells stained positive for S100 and HMB-45. The patient underwent a course of interferon-α therapy (5 million/IU/day) for two weeks after surgery; treatment was then discontinued after one month due to fatigue and weight loss [3]. In the second case, the patient was a 38-year-old woman who presented with pain in the left breast and axillary region. The skin examination was unremarkable and no ophthalmologic examination was completed. The patient's laboratory studies, including a urinalysis, were within normal limits. A chest X-ray showed a suspicious mass in the right middle lobe of the lung, and a follow-up CT scan showed two lung masses and a solid, enhancing lesion in the left kidney. Biopsy of the lung mass confirmed the diagnosis of melanoma as the cells stained positive for melan A. The patient then underwent a left radical nephrectomy and pathology showed histology identical to the lung mass, indicating the patient had MUP. For treatment, the patient underwent a high-dose course of interleukin-2 (IL-2) and showed a 40% reduction in tumor volume of the lung mass. She did start a second course of IL-2, as well [2]. Interestingly, when comparing patient presentations, our patient presented with gastrointestinal symptoms. This highlights the importance of a comprehensive work-up in terms of diagnosing MUP. 

**Table 1:** Comparison of Reported Cases of Melanoma of Unknown Primary Presenting as a Renal Mass CT - computed tomography; PET - positron emission tomography

Characteristic	Current Case	Case 1 [3]	Case 2 [2]
Age/Sex	66-year-old male	67-year-old male	38-year-old female
Presenting Symptoms	Weight loss and gastrointestinal distress	Right flank pain and microscopic hematuria	Left breast and axillary pain
Renal Laterality	Right	Left	Left
Renal Location	Superior pole, exophytic	Lower pole	Not reported
Extra-renal Disease at Diagnosis	No	No	Lung metastases present
Initial Detection	CT scan	Ultrasound	CT scan
Primary Lesion Identified	No	No	No
Workup for Primary Lesion	Dermatologic, ophthalmologic, urologic evaluations; bladder and prostatic urethral biopsies	Dermatologic and ophthalmologic evaluations	Dermatologic evaluation
Surgical Management	Robotic right partial nephrectomy	Surgical resection (not specified)	Left radical nephrectomy
Histopathology/Immunohistochemistry	Positive for Sox-10, Mart-1, HMB-45	Positive for S100 and HMB-45	Positive for Melan-A
Treatment	Ipilimumab and nivolumab, followed by nivolumab maintenance	Interferon-α (discontinued due to side effects)	High-dose interleukin-2
Clinical Outcome	No evidence of disease on recent PET CT scan	Not reported	Partial response, ≈40% reduction in tumor burden

There are several hypotheses to explain the phenomenon of MUP, including spontaneous regression, ectopic melanocytes, and differentiation of pre-existing stem cells in lymph nodes or visceral organs [1]. The overall incidence of MUP is 3.2%, according to a systematic review completed in 2011 [6]. MUP is twice as likely to occur in men and most commonly occurs in cervical, axillary, inguinal, and parotid lymph nodes [1]. Additionally, MUP metastatic to lymph nodes was more common in men, where metastases to the extremities were more common in women [6]. 

Regarding prognostic factors, median overall survival for MUP patients with lymph node involvement (AJCC stage III) ranges from 24 months to 127 months [1]. Overall survival for MUP patients with visceral involvement has a worse prognosis of three to 13 months [1]. Furthermore, studies have shown contradicting results on whether MUP has a better survival rate compared to those with primary melanoma [3]. Two different studies demonstrated that MUP had a better survival rate compared to patients with advanced and metastatic primary cutaneous melanoma [7,8]. 

It is important to distinguish MUP from primary non-cutaneous melanoma, which arise from melanocytes as ocular or mucosal melanomas [4]. Mucosal melanomas may develop at any site with a mucosal membrane lining, including respiratory, gastrointestinal, and urogenital tracts [4]. Occasionally, primary non-cutaneous melanoma can be misclassified as MUP due to an incomplete physical examination or inadequate diagnostic workup [1]. Therefore, before establishing a diagnosis of MUP, patients must undergo extensive evaluation, including dermatologic, ophthalmologic, and urologic examinations, to identify a potential primary lesion as was performed in our patient. Genetic analyses further support this distinction, demonstrating that MUP shares a similar genetic profile to cutaneous melanoma through BRAF and Neuroblastoma RAS Viral Oncogene Homolog (NRAS) mutations, whereas primary non-cutaneous melanoma demonstrates more frequent c-KIT mutations [1]. 

Surgical resection of the tumor metastasis, often the lymph nodes, is essential to improve patient survival. Additionally, oncological treatment, such as chemotherapy, radiotherapy, or immunotherapy, should always follow surgical resection [3]. Novel immunotherapy regimens include Cytotoxic T-Lymphocyte Associated Protein 4 (CTLA-4) antibodies, ipilimumab, and Programmed cell death protein 1 (PD-1) receptor antibodies, nivolumab, and pembrolizumab. A phase 2 clinical trial in 2023 found neoadjuvant pembrolizumab followed by adjuvant pembrolizumab therapy to significantly increase event-free survival at two years by 23% [9]. Stage IV MUP requires more extensive and aggressive therapy, including surgical resection, immunotherapy, chemotherapy, and/or radiotherapy.

## Conclusions

MUP is a rare presentation of melanoma and it is exceedingly uncommon to be discovered in the kidney. According to the present literature, there have only been two other cases of MUP in the kidney identified. Considering MUP as a differential diagnosis in renal masses is crucial, especially when patients present without cutaneous or ocular melanotic lesions. Early detection due to the aggressiveness of this tumor and the low survival rates is important in providing the patient with prompt treatment.
